# Case Report: Brainstem angiocentric glioma presenting in a toddler child–diagnostic and therapeutic challenges

**DOI:** 10.3389/pore.2023.1611231

**Published:** 2023-06-09

**Authors:** Zita Reisz, Bence Laszlo Radics, Peter Nemes, Ross Laxton, Laszlo Kaizer, Krisztina Mita Gabor, Timea Novak, Pal Barzo, Safa Al-Sarraj, Istvan Bodi

**Affiliations:** ^1^ Department of Clinical Neuropathology, King’s College Hospital, London, United Kingdom; ^2^ Department of Pathology, University of Szeged, Szeged, Hungary; ^3^ Department of Neurosurgery, University of Szeged, Szeged, Hungary; ^4^ Department of Pediatrics and Pediatric Healthcare Center, University of Szeged, Szeged, Hungary; ^5^ Department of Radiology, University of Szeged, Szeged, Hungary

**Keywords:** paediatric brainstem glioma, angiocentric glioma, *MYB:QKI* fusion, DNA methylation profiling, RNA sequencing

## Abstract

**Introduction:** Angiocentric gliomas (AG) in brainstem location are exceedingly rare and might cause differential diagnostic problems and uncertainty regarding the best therapeutic approach. Hereby, we describe the clinicopathological findings in a brainstem AG presenting in a toddler child and review the literature.

**Case report:** A 2-year-old boy presented with 5 weeks history of gait disturbances, frequent falls, left-sided torticollis and swallowing problems. MRI head showed a T2-hyperintense, partly exophytic mass lesion centred in the pontomedullary region, raising the possibility of diffuse midline glioma. The exophytic component was partially resected by suboccipital craniotomy, leaving intact the infiltrative component. Ventriculoperitoneal shunt was implanted due to postoperative hydrocephalus. Histological examination revealed a moderately cellular tumour consisted of bland glial cells infiltrating the brain parenchyma and radially arranged around the blood vessels. By immunohistochemistry, the tumour strongly expressed S100 and GFAP in addition to intense nestin positivity, while OLIG2 was negative in the perivascular tumour cells. DNA methylation array profiled the tumour as “methylation class diffuse astrocytoma, *MYB* or *MYBL1*-altered subtype B (infratentorial)” and an in-frame *MYB::QKI* fusion was identified by RNA sequencing, confirming the diagnosis of angiocentric glioma. The patient has been initially treated with angiogenesis inhibitor and mTOR inhibitor, and now he is receiving palliative vinblastine. He is clinically stable on 9 months follow-up.

**Conclusion:** Brainstem AG may cause a diagnostic problem, and the surgical and oncological management is challenging due to unresectability and lack of response to conventional chemo-radiation. In the future, genetically-tailored therapies might improve the prognosis.

## Introduction

Paediatric brainstem gliomas are diagnostically challenging as in most cases only stereotactic biopsy is safely amenable and the histological features might not be entirely representative in a small sample. Tumours arising from the midbrain, pontomedullary area or the fourth ventricle encompass a molecularly heterogeneous group of disorders, the most common entities are being pilocytic astrocytoma, diffuse midline glioma (DMG), H3 K27-altered, posterior fossa ependymoma, and medulloblastoma. Other circumscribed or diffuse astrocytomas and glioneuronal tumours can also rarely present in this location, many of them driven by MAPK pathway or FGFR alterations with closely clustered methylation profiles, causing further diagnostic difficulties in limited samples [1–3].

Angiocentric glioma (AG) was first described in 2005 by Wang and was originally being categorized under the umbrella of “other neuroepithelial tumours” in the 2007 WHO Classification of the Central Nervous System Tumours due to its uncertain histogenesis [4, 5]. AG is typically stable or slow growing, consisting of monomorphous bipolar cells with a predominant diffuse growth pattern and at least focal perivascular tumour cell aggregation around the blood vessels (the so-called angiocentric pattern). Dot-like paranuclear EMA positivity is a characteristic feature, representing microlumina formation similarly to ependymomas [4, 5]. Molecularly, most tumours were found to harbour a *MYB::QKI* fusion, and the remaining cases are usually associated with other *MYB* or *QKI* alterations [6, 7].

AGs most commonly involve supratentorial cortex, and particularly the frontoparietal and temporal lobes, presenting with long-standing therapy refractory epilepsy with a median age at the diagnosis is 13 years [4, 8–10]. The clinical course is indolent and good seizure control can be achieved with total surgical resection. Exceedingly rare cases in the brainstem have been reported with similar morphological features and molecular profile to their supratentorial counterparts, but data regarding their clinical behaviour and the optional therapeutic approaches are limited, particularly under age of 3 years [11–14].

Here, we describe the clinicopathological features and the molecular findings in a brainstem AG presenting in a 2-year-old boy and review the literature for treatment options.

## Clinical summary

**FIGURE 1:**
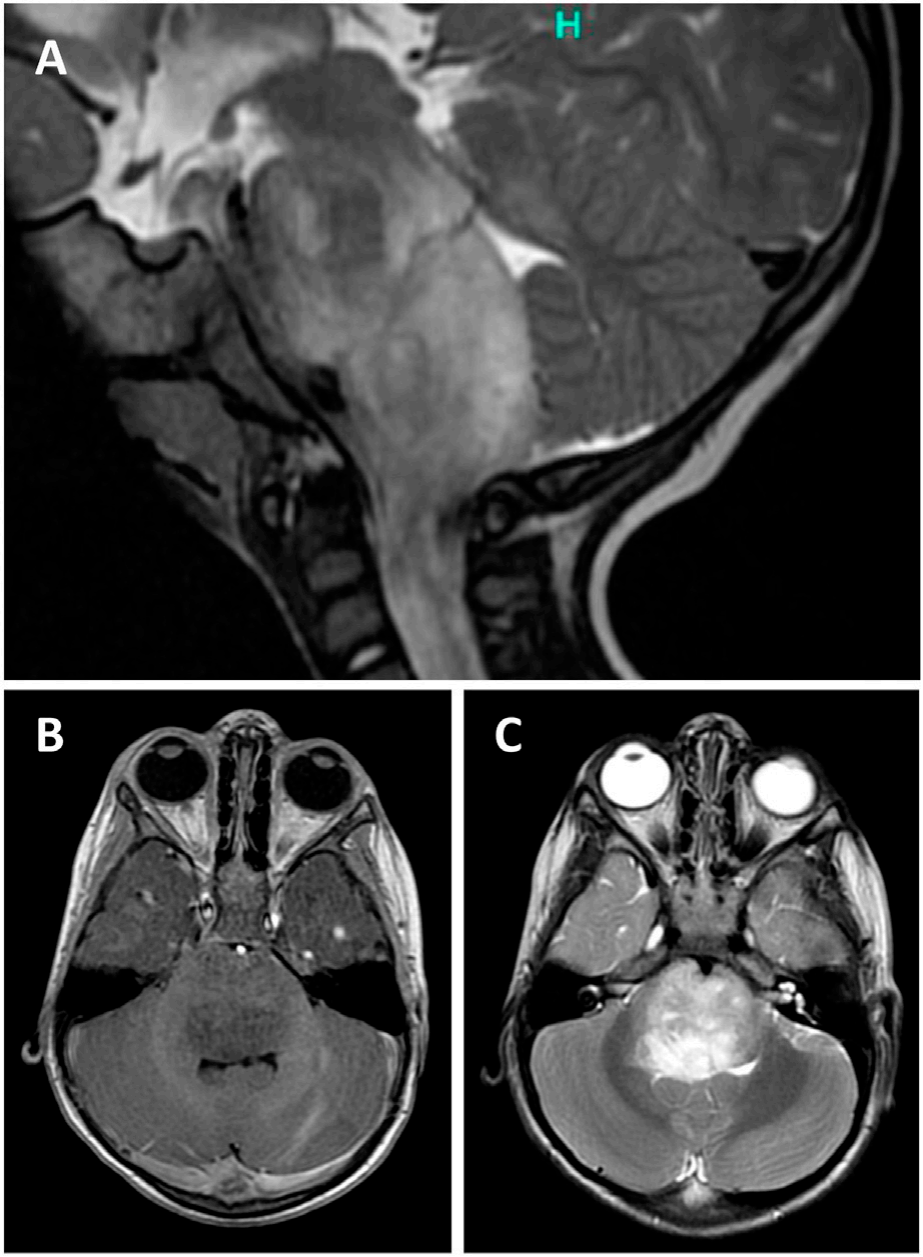
Sagittal T2-weighted (T2W) magnetic resonance image (MRI) of the posterior fossa showing a partly exophytic mass lesion in the pontine and medullary regions with 30 mm axial and 49 mm cranio-caudal greatest extension **(A)**. The tumour is hypointense on T1W **(A)** and hyperintense on T2W **(B)** imaging without significant contrast enhancement.

Two-year-old boy presented with 5 weeks history of gait disturbances, frequent falls and a recently developed predominant left-sided neck position. He also had difficulties with swallowing and has been less communicative lately. There was no history of fever and vomiting. His past medical history was not significant. On admission, neurology examination revealed cerebellar ataxia and right abducens nerve palsy. MRI head showed a partly exophytic mass lesion in the pontine and medullary regions with 31 × 30 mm axial and 49 mm cranio-caudal greatest extension (Figure 1). The tumour was hypointense on T1-weighted and hyperintense on T2-weighted imaging without significant contrast enhancement and negative on diffusion weighted imaging. Clinically diffuse midline glioma was suspected and a partial resection was performed by suboccipital craniotomy and intraoperative neuronavigation, removing some exophytic mass and leaving intact the infiltrative brainstem component. Recovery was achieved at postoperative day 4, remaining dysphagic and tracheostomized. On postoperative day 11, he became drowsy and urgent MRI confirmed moderate hydrocephalus. A ventriculo-peritoneal shunt was implanted the following day which needed revision due to shunt infection. Oncological treatment began 1 month after the surgery with angiogenesis inhibitor (bevacizumab) and mTOR inhibitor (temsirolimus). Follow up MRI at 4 months after surgery showed minimal growth in axial dimension (32 × 37 mm), while the cranio-caudal dimension did not change. He is receiving palliative vinblastine. The patient remains clinically stable 9 months after surgery. A timeline is attached in Supplementary Figure S1 showing the treatment regime and the most important clinical events in the patients clinical history.

## Pathological findings

**FIGURE 2:**
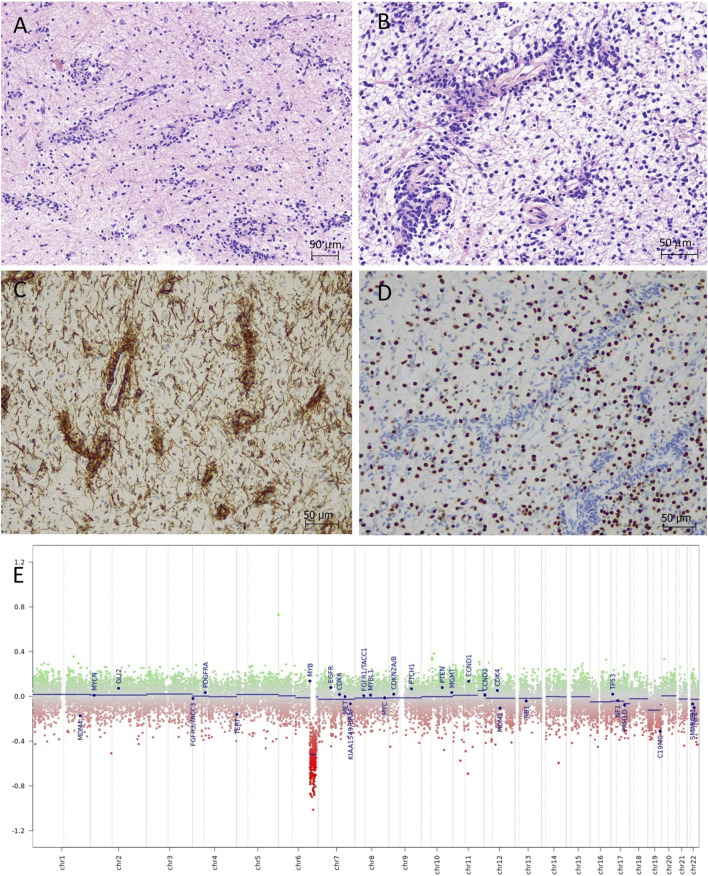
Angiocentric glioma (haematoxylin-eosin). **(A,B)** Moderately cellular tumour with perivascular tumour cell aggregation of radially arranged mono- and multi-layered rosettes around all blood vessels. The intervascular spaces contained loosely arranged bipolar cells embedded in a microcystic matrix. **(C)** The perivascular tumour cells are strongly positive with nestin by immunohistochemistry. **(D)** OLIG2 is positive only in the intervascular cells and negative in the perivascular tumour cells. **(E)** Copy number variation plot by DNA methylation array shows segmental loss of chromosome 6q.

Histological sections showed small tumour fragments in places infiltrating brainstem parenchyma (Figure 2A). The tumour had low to moderate cellularity and consisted of monomorphic glial cells with small, elongated nuclei and indiscernible cytoplasm. The dominant feature was the striking perivascular tumour cell aggregation which created characteristic mono and multi-layered rosettes virtually around all blood vessels (angiocentric pattern), while the intervascular spaces contained loosely arranged cells embedded in a microcystic matrix and intermingled with entrapped otherwise normal-looking neurons (Figure 2B). There were no Rosenthal fibres, eosinophilic granular bodies or dysplastic ganglion cells seen. Mitotic figures were not identified and there was no necrosis or microvascular proliferation. By immunohistochemistry (Supplementary Table S1), the tumour strongly expressed S100 and GFAP in addition to intense nestin positivity (Figure 2C). The synaptophysin labelled mainly the background brain parenchyma and was negative within the tumour. OLIG2 was negative in the perivascular tumour cells but expressed in most intervascular cells (Figure 2D). There was focal paranuclear dot-like EMA positivity, particularly in the perivascular areas (Supplementary Figure S2). The mutant IDH1 (R132H) and H3 K27M were negative. The nuclear expression was retained for H3 K27me3 and ATRX. The p53 was negative. The Ki67 proliferation index was estimated at 3%–4%. The histological features and the initial immunoprofile were strongly suggestive of brainstem AG; however, the possibility of other rare molecularly defined paediatric-type diffuse low-grade glioma/glioneuronal tumours remained in the differential diagnosis.

DNA methylation array by Illumina MethylationEPIC 850k (DKFZ Brain tumour methylation classifier v12.5) confidently profiled the tumour as “methylation class diffuse astrocytoma, *MYB* or *MYBL1*-altered, subtype B [infratentorial]” (calibrated score of 0.99906), which is a provisional methylation subclass awaiting further specification (Molecular methods provided in Supplementary Material). Copy number variation plot generated from methylation array data showed segmental loss of chromosome 6q with no obvious involvement of the *MYB* locus (Figure 2E); however, possible deletion at 6q23.3 (*MYB* locus) was suspected by Integrative Genomics Viewer (IGV). The array predicted unmethylated *MGMT* gene promoter. Next-generation sequencing (Qiagen QIAseq Multimodal Panel assessing a targeted DNA panel of 305 genes and an RNA panel of 76 genes associated with solid tumours) found an in-frame *MYB::QKI* fusion between *MYB* exon 15 and *QKI* exon 5 with retained C-terminal regulatory, LMSTEN motif and Myb-like DNA-binding domains and loss of the 3′UTR regulatory site. There were no additional pathogenic variants seen by DNA panel. The integrated diagnosis was given as “Angiocentric glioma, CNS WHO grade 1.”

## Discussion

According to the fifth edition of the WHO classification of Central Nervous System Tumours, AG is defined as a diffuse glioma comprising cytologically bland, bipolar cells aggregating at least partly in perivascular spaces and typically harbouring *MYB*::*QKI* gene fusion or other *MYB* alteration, corresponding to CNS WHO grade 1 [15]. The most common location is the cerebral cortex, manifesting with early onset of seizures, hence it belongs to “low-grade epilepsy-associated neuroepithelial tumours (LEAT)” according to the International League against of Epilepsy (ILAE) [8]. These tumours remain stable in size over the years and can be controlled by anti-epileptic drugs to a certain degree, and are potentially curable with gross total resection (GTR) [16].

AGs arising in brainstem are exceedingly rare and have been described only in isolated case reports and small case series with a total of 15 cases to date (Table 1). Patients typically present at a younger age, approximately 10–15 years earlier than in supratentorial location (median age at 4 years) and they often have a shorter clinical history with recently developed and progressive symptoms due to advanced disease stage. On radiology, AG present with high intensity on T2-weighted and fluid-attenuated inversion recovery (FLAIR) images and low intensity on T1-weighted image usually without contrast enhancement [10, 19]. Intratumoral calcification or haemorrhage is uncommon. Cortical lesions may extend to the subcortical white matter and sometimes stalk-like extension to the ventricle also occurs. In brainstem location, differential diagnosis of DMG may be raised, as in our case, although the latter may show contrast enhancement.

**TABLE 1:** Summary of clinical and molecular data of published brainstem angiocentric gliomas.

References	Sex	Age	Methylation array	FISH/RNA fusion	Surgery	Chemotherapy/Radiation	Survival/Follow-up
[12]	F	5	no	not tested	debulking	no	stable residual after 2 years follow-up
[14]	F	5	no	not tested	biopsy, ETV	carboplatin	adjuvant chemotherapy, progression at 6 months, near total tumour resection; minimal stable residual after 6 years follow-up
[14]	M	6	no	not tested	biopsy, ETV	no	stable after 1.5 years follow-up
[13]	M	7	no	MYB::QKI fusion	biopsy	carboplatin and vincristine; later vinblastin and bevacizumab	clinical progression after 4 months following treatment, stable MRI
[13]	M	3	no	MYB::QKI fusion	biopsy	carboplatin, vincristine	mTOR inhibitor (Everolimus) due progression after 3 years–stable after 10 months
[11]	M	7	no	MYB::QKI fusion	biopsy	no data	no data
[17]	F	4	no	MYB rearrangement	biopsy	radiation	stable disease; follow-up NA
[17]	F	4	no	MYB rearrangement	biopsy	radiation	stable disease; follow-up NA
[17]	M	3	yes	MYB::QKI fusion	biopsy	yes	stable disease; follow-up NA
[17]	F	2	yes	MYB::QKI fusion	biopsy	radiation	stable disease; follow-up NA
[17]	F	1	no	MYB rearrangement	biopsy	chemoradiation	progression (50 months follow-up)
[17]	F	4	yes	MYB rearrangement	biopsy	no data	stable disease; follow-up NA
[17]	F	6	no	MYB	biopsy	no data	stable disease; follow-up NA
[18]	F	4	no	not tested	debulking	Carboplatin, Vincristine	progression after 7 years; palliative therapy
[19]	M	2	no	not tested	biopsy	yes	survive (39 months follow-up)
Current case	M	2	yes	MYB::QKI fusion	debulking, ETV	bevacizumab, temsirolimus	stable (4 months follow-up)

M, male; F, female; ETV, endoscopic third ventriculostomy; NA, not available.

Pathologically, the differential diagnosis is broad and often challenging, particularly in intraoperative smears and small biopsies. Before the molecular era, the most common misdiagnosis was pilomyxoid astrocytoma due to similar cellular morphology and perivascular accentuation of tumour cell, however, the negative or only focal OLIG2 staining and paranuclear dot-like EMA positivity should warrant the diagnosis of brainstem AG [17]. Angiocentric pattern might be also mistaken for ependymal pseudorosettes, further complicated by their overlapping immunoprofiles, but the elongated nuclear shape, absence of ependymal rosettes or canals together with strong nestin positivity would argue against ependymoma. Some tumours exhibit solid schwannoid growth pattern although the cytological monomorphism is against vestibular schwannoma. Diffuse midline glioma can be easily excluded by immunopanel for surrogate molecular markers including tri-methylated histone H3 K27 (H3 K27me3), mutant H3 K27M, and EZHIP. The diagnosis can rely on DNA methylation profiling, as these tumours form a clearly distinct methylation cluster including novel infratentorial subclasses, probably reflecting slightly different cellular origin for supra- and infratentorial tumours [20].

Regardless the location of the tumour, the driver molecular alteration in majority of the cases is a *MYB::QKI* rearrangements [6, 19]. The proto-oncogen *c-MYB* is a transcriptional regulator playing an important role in neural progenitor cell proliferation and found to be expressed mainly in the ependymal/sub-ventricular zone in a mature brain [20]. The protein consists of highly conserved DNA motifs including an N-terminal DNA binding motifs followed by a transcriptional activation domain and a C-terminal negative regulatory domain. *MYB::QKI* rearrangements with breakpoints within intron 4 of *QKI* and intron 9–15 of *MYB* creates an in-frame aberrant fusion protein with loss of 3′UTR regulatory (miRNA binding) site or the whole C-terminal domain, eventually leading to expression of a truncated (oncogenic) *MYB*. Activation of the pathway might be further increased by the relocation of the H3 K27ac enhancer elements in close proximity to MYB promoter resulting in an independent auto-regulatory circle. Rarely, *MYB* gene can have alternative fusion partners like *ESR1* or the pathway can be upregulated as a consequence of *MYB* amplification, but *QKI* rearrangement with no obvious *MYB* or *MYBL1* involvement has been reported as well [6, 7]. This supports the idea that disruption of the *QKI* tumour suppressor gene also plays a key role in the tumorigenesis in angiocentric gliomas [6]. Concomitant mutations in MAPK pathway, particularly *BRAF V600E* mutation, was found in isolated cases [7].

Brainstem AGs require different surgical and neuro-oncological management compared to their supratentorial counterparts. While complete resection tends to be curative for tumours in the cortex and it usually results in good seizure control, gross total resection in the brainstem is not feasible because of often widespread involvement of the key anatomical structures [10–14, 17, 18]. Stereotactic biopsy is the chosen technique in most cases to get tissue sample for diagnostic purposes, while symptoms of ventricular obstruction can be managed by debulking of the exophytic component in addition to endoscopic ventriculostomy (ETV). The extent of surgical resection was found to be a prognostic factor; however, the significant residual tumour, present in most cases, may eventually progress similarly to other brainstem low-grade gliomas [11–14, 17, 18]. Most frequent complications reported in the literature was the obstructive hydrocephalus, brainstem dysfunction and cranial nerve abnormalities, all of which had a significant effect on the quality of the patient’s life. Due to limited surgical options, current therapy may vary between close observation and adjuvant chemotherapy and/or radiotherapy. According to the literature on previously reported brainstem AGs, conventional chemotherapy (mainly vincristine and carboplatin) were ineffective; while application of radiotherapy should be carefully considered under age of 3 years due to its devastating effect to the developing brain [13, 14, 17, 18]. In one case, after fail of conventional chemo agents, the therapy was changed to mTOR inhibitor (everolimus) with good initial tumour response and stable disease after 10 months on follow-up [13, 21]. In the future, genomically tailored therapies targeting the activated MYB pathway or modifying its upstream regulatory elements might be an option to improve patient management; however, this needs to be validated through clinical trials.

## Conclusion

Angiocentric glioma of the brainstem is rare and needs to be considered as a distinct clinicopathological entity due to its less favourable outcome often associated with significant decrease of quality of life. In limited samples, the pathological diagnosis might be challenging and requires molecular conformation by DNA methylation profiling and high-throughput sequencing techniques including a large-scale RNA fusion panel. Unfortunately, therapeutic options are limited due to low tumour respectability in the eloquent location, and conventional chemoradiotherapy appears ineffective to halt tumour progression over the years which highlights the importance of future development of individualised therapies targeting cancer pathways involved in gliomagenesis.

## Data Availability

Access to the full data used in the study is available from the authors upon request.

## Abbreviations

ATRX, alpha-thalassemia/mental retardation, X-linked; AG, angiocentric glioma; DMG, diffuse midline glioma; EMA, epithelial membrane antigen; EZHIP, EZH inhibitory protein; FLAIR, fluid-attenuated inversion recovery; FGFR, fibroblast growth factor receptor; GFAP, glial fibrillary acidic protein; GTR, gross total resection; IDH1, isocytrate dehydrogenase 1; ILAE, international league against of epilepsy; LEAT, low-grade epilepsy-associated neuroepithelial tumours; MAPK, mitogen-activated protein kinase; MRI, magnetic resonance imaging, mTOR, mammalian target of rapamycin; MYB, myeloblastosis; MYBL1, MYB proto-oncogene Like 1; OLIG2, oligodendrocyte transcription factor; QKI, quaking homolog; KH, domain RNA binding; WHO, world health organisation.

## References

[1] Chen LH Pan C Diplas BH Xu C Hansen LJ Wu Y The integrated genomic and epigenomic landscape of brainstem glioma. Nat Commun (2020) 11(1):3077–11. 10.1038/s41467-020-16682-y 32555164 PMC7299931

[2] Ryall S Tabori U Hawkins C. Pediatric low-grade glioma in the era of molecular diagnostics. Acta Neuropathol Commun (2020) 8(1):30–22. 10.1186/s40478-020-00902-z 32164789 PMC7066826

[3] Ryall S Zapotocky M Fukuoka K Nobre L Guerreiro Stucklin A Bennett J Integrated molecular and clinical analysis of 1,000 pediatric low-grade gliomas. Cancer Cell (2020) 37(4):569–83. 10.1016/j.ccell.2020.03.011 32289278 PMC7169997

[4] Wang M Tihan T Rojiani AM Bodhireddy SR Prayson RA Iacuone JJ Monomorphous angiocentric glioma: A distinctive epileptogenic neoplasm with features of infiltrating astrocytoma and ependymoma. J Neuropathol Exp Neurol (2005) 64(10):875–81. 10.1097/01.jnen.0000182981.02355.10 16215459

[5] Louis DN Ohgaki H Wiestler OD Cavenee WK Burger PC Jouvet A The 2007 WHO classification of tumours of the central nervous system. Acta Neuropathol (2007) 114(2):97–109. 10.1007/s00401-007-0243-4 17618441 PMC1929165

[6] Bandopadhayay P Ramkissoon LA Jain P Bergthold G Wala J Zeid R MYB-QKI rearrangements in angiocentric glioma drive tumorigenicity through a tripartite mechanism. Nat Genet (2016) 48(3):273–82. 10.1038/ng.3500 26829751 PMC4767685

[7] Qaddoumi I Orisme W Wen J Santiago T Gupta K Dalton JD Genetic alterations in uncommon low-grade neuroepithelial tumors: BRAF, FGFR1, and MYB mutations occur at high frequency and align with morphology. Acta Neuropathol (2016) 131(6):833–45. 10.1007/s00401-016-1539-z 26810070 PMC4866893

[8] Blümcke I Aronica E Becker A Capper D Coras R Honavar M Low-grade epilepsy-associated neuroepithelial tumours — The 2016 WHO classification. Nat Rev Neurol (2016) 12(12):732–40. 10.1038/nrneurol.2016.173 27857123

[9] Lellouch-Tubiana A Boddaert N Bourgeois M Fohlen M Jouvet A Delalande O Angiocentric neuroepithelial tumor (ANET): A new epilepsy-related clinicopathological entity with distinctive MRI. Brain Pathol (2005) 15(4):281–6. 10.1111/j.1750-3639.2005.tb00112.x 16389940 PMC8095937

[10] Preusser M Hoischen A Novak K Czech T Prayer D Hainfellner JA Angiocentric glioma: Report of clinico-pathologic and genetic findings in 8 cases. Am J Surg Pathol (2007) 31(11):1709–18. 10.1097/PAS.0b013e31804a7ebb 18059228

[11] Chan E Bollen AW Sirohi D Van Ziffle J Grenert JP Kline CN Angiocentric glioma with MYB-QKI fusion located in the brainstem, rather than cerebral cortex. Acta Neuropathol (2017) 134(4):671–3. 10.1007/s00401-017-1759-x 28776091 PMC5693679

[12] Covington DB Rosenblum MK Brathwaite C Sandberg DI. Angiocentric glioma-like tumor of the midbrain. Pediatr Neurosurg (2010) 45:429–33. 10.1159/000277616 20110754

[13] D’Aronco L Rouleau C Gayden T Crevier L Décarie J-C Perreault S Brainstem angiocentric gliomas with MYB-QKI rearrangements. Acta Neuropathol (2017) 134(4):667–9. 10.1007/s00401-017-1763-1 28803398 PMC6556888

[14] Weaver KJ Crawford LM Bennett J Rivera-Zengotita ML Pincus DW. Brainstem angiocentric glioma: Report of 2 cases. J Neurosurg Pediatr (2017) 20(4):347–51. 10.3171/2017.5.PEDS16402 28753090

[15] Louis DN Perry A Wesseling P Brat DJ Cree IA Figarella-Branger D The 2021 WHO classification of tumors of the central nervous system: A summary. Neuro Oncol (2021) 23(8):1231–51. 10.1093/neuonc/noab106 34185076 PMC8328013

[16] Han G Zhang J Ma Y Gui Q Yin S. Clinical characteristics, treatment and prognosis of angiocentric glioma. Oncol Lett (2020) 20(2):1641–8. 10.3892/ol.2020.11723 32724405 PMC7377082

[17] Chiang J Harreld JH Tinkle CL Moreira DC Li X Acharya S A single-center study of the clinicopathologic correlates of gliomas with a MYB or MYBL1 alteration. Acta Neuropathol (2019) 138:1091–2. 10.1007/s00401-019-02081-1 31595312 PMC7467132

[18] Almubarak AO Alahmari A Al Hindi H AlShail E. Angiocentric glioma of brainstem. Neurosciences (2020) 25(5):416–20. 10.17712/nsj.2020.5.20200026 33459294 PMC8015589

[19] Kurokawa R Baba A Emile P Kurokawa M Ota Y Kim J Neuroimaging features of angiocentric glioma: A case series and systematic review. J Neuroimaging Off J Am Soc Neuroimaging (2022) 32(3):389–99. 10.1111/jon.12983 PMC9306893 35201652

[20] Capper D Stichel D Sahm F Jones DTW Schrimpf D Sill M Practical implementation of DNA methylation and copy-number-based CNS tumor diagnostics: The heidelberg experience. Acta Neuropathol (2018) 136(2):181–210. 10.1007/s00401-018-1879-y 29967940 PMC6060790

[21] Ho AL Vasudeva SD Schwartz GK. Abstract 638: The impact of PI3K/Akt pathway inhibition upon the c-myb oncogene. Cancer Res (2011) 15;638. 10.1158/1538-7445.AM2011-638

