# Supplementary material for: Case Report: Brainstem angiocentric glioma presenting in a toddler child–diagnostic and therapeutic challenges
Source: Pathol Oncol Res. 2023 Jun 9;29:1611231. doi: 10.3389/pore.2023.1611231 (PMC10287963; doi:10.3389/pore.2023.1611231)
Supplement: Supplementary file 1 [file DataSheet2.pdf]

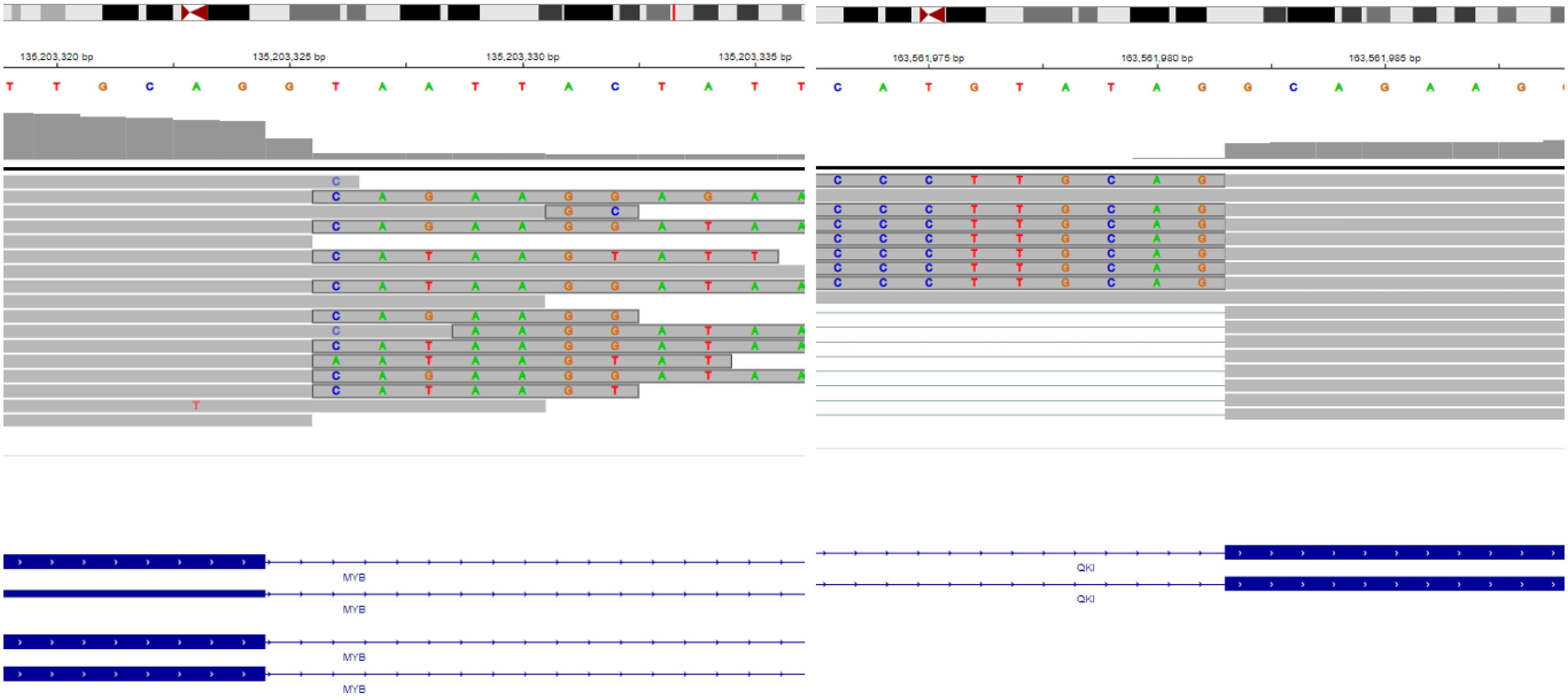

Supplementary figure 3. The Integrative Genomics Viewer (IGV) showing the “soft-clipped” reads that match the corresponding genes demonstrating an in-frame *MYB::QKI* fusion.
