# Supplementary material for: Case Report: Brainstem angiocentric glioma presenting in a toddler child–diagnostic and therapeutic challenges
Source: Pathol Oncol Res. 2023 Jun 9;29:1611231. doi: 10.3389/pore.2023.1611231 (PMC10287963; doi:10.3389/pore.2023.1611231)
Supplement: Supplementary file 2 [file DataSheet1.pdf]

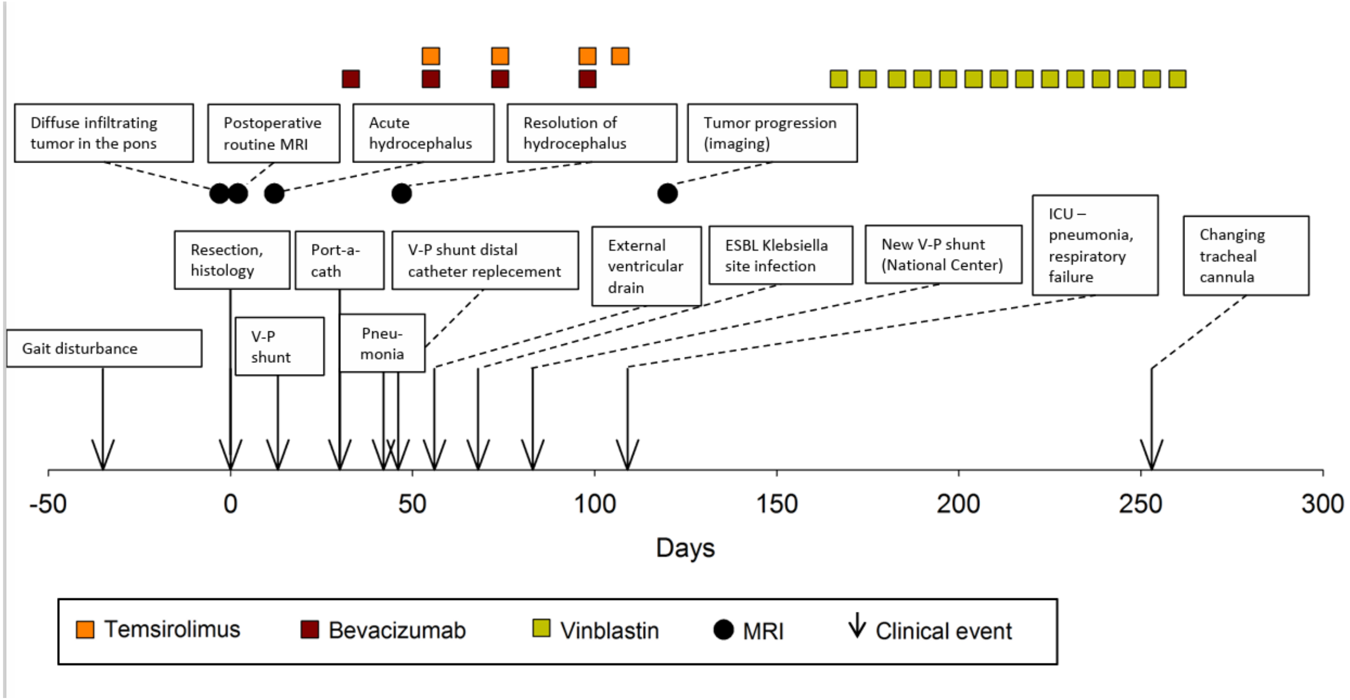

Supplementary figure 1. Timeline of treatment regime and clinical events.

Antibody	Manufacturer (clone)	Dilution
GFAP	BioSB (G-A-5)	1:2000
EMA	Cellmarque (E29)	1:2000
Nestin	Santa Cruz (10C2)	1:200
Synaptophysin	Novocastra (27G12)	1:400
OLIG2	Abcam (EPR2673)	1:50
IDH1 (R132H)	GenomeMe (IHC132)	1:200
ATRX	Sigma-Aldrich (polyclonal)	1:300
p53	Cellmarque (DO7)	1:300
Ki-67	Hisztopathologia (SP6)	1:100
S100	Cellmarque (4C4.9)	1:800
H3 K27M	Millipore (polyclonal)	1:800
H3 K27me3	Cell Signaling (C36B11)	1:200

Supplementary table 1. Antibodies, manufacturers, and dilutions used for immunohistochemistry.

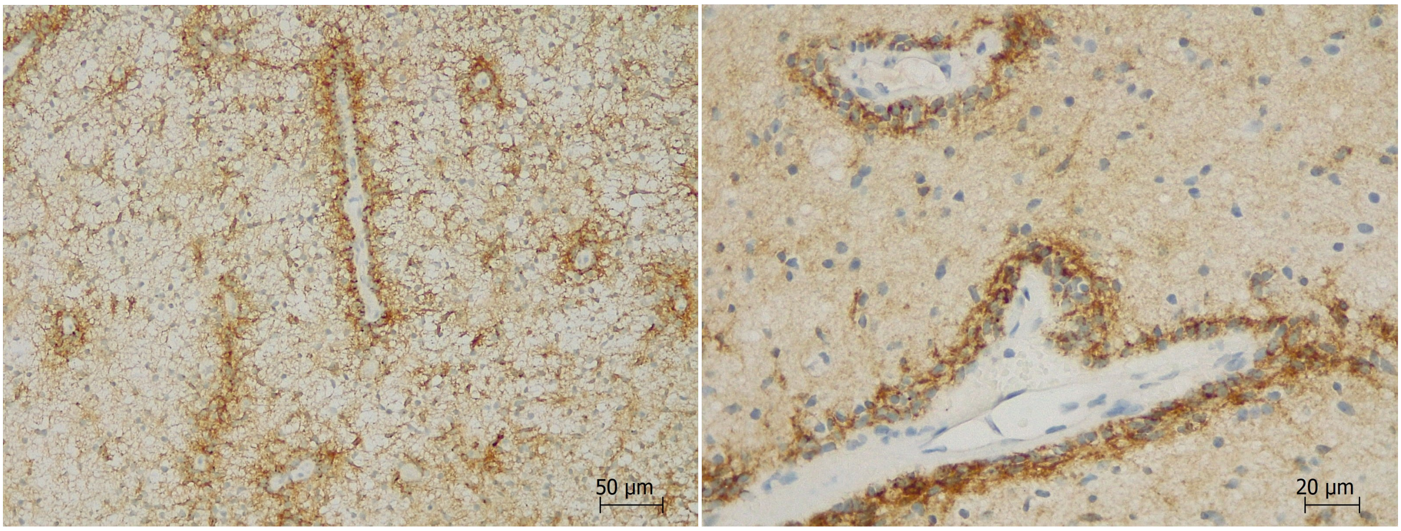

Supplementary figure 2. Immunohistochemistry for EMA showing focal paranuclear dot-like EMA positivity, particularly in the perivascular areas.

Methods for the molecular studies

1. DNA and RNA extraction

AllPrep DNA/RNA FFPE Kit were used on the QIAcube Connect MDX. DNA and RNA were extracted from Formalin-fixed paraffin embedded (FFPE) sample.

2. Methylation Array

a) Samples bisulfite converted using the Zymo EZ DNA Methylation™ Kit (D5001 or D5002), FFPE samples are restored using Illumina Restore Kit (WG-321-1002) as per manufacturer's protocols. Bisulfite treated samples are processed using the Illumina Infinium Methylation EPIC Kit which analyses 850k methylation sites (as per manufacturer's protocols).
b) Diagnostic methylation array testing using the Illumina Infinium HD Methylation Array. Samples are scanned on an Illumina iSCAN. Data is processed via the Molecular Neuropathology (MNP) classifier developed and hosted by the German Cancer Research Centre in Heidelberg (www.molecularneuropathology.org/mnp). Methylation class calibration scores >0.9 are reported with high confidence. Calibration scores between 0.3 and 0.9 are reported with low confidence. Calibration scores <0.3 are considered a failed classification.
c) CNVs are called by the conumee package[2] and visual inspection. CNVs described are relative to baseline, copy numbers may vary dependent on ploidy of the tumour sampled. log2 values +0.15 to +0.4 indicate gains, values >+0.4 indicate amplification. log2 values -0.15 to -0.4 indicate loss, values <-0.4 indicate homozygous loss. Lower level CNVs may be reported following visual inspection.
lecular Pathology." *Genetics in medicine* 17, no. 5 (2015): 405-423.

3. DNA and RNA-Fusion Targeted sequencing

Diagnostic testing of a panel of genes associated with the relevant clinical phenotype described in the national genomic test directory indication, for patients meeting the eligibility criteria (national-genomic-test-directory-cancer-2021-22-v2-december-2021).

a) Testing of the indicated gene(s) is by the Qiagen QIAseq Multimodal Panel, using single primer extension with unique molecular indexes to assess a targeted DNA panel of 305 genes and a RNA panel of 76 genes associated with solid tumours. Additional virtual DNA panels may be applied as required, please contact the laboratory to discuss. Samples are sequenced on an Illumina NextSeq2000, 2X150bp reads with a minimum coverage of 400x (minimum of 6 reads to make a mutation call). Variants at <2% are excluded from analysis and variants at <5% are not considered clinically significant and are therefore not reported. Read alignment and variant calling is performed by BWA-MEM, Samtools, Picard and Vardict followed by BcfTools for variant filtering. This test has been shown to have a minimum sensitivity of 98.3% for single nucleotide substitutions and small insertion/deletion variants for regions covered by >=400x with a 95% confidence interval. Somatic variants with allelic frequencies <0.1, or in areas of low coverage, will be at significantly higher risk of not being detected. The sensitivity for detection of SVs and CNVs is yet to be determined, though it is likely to be lower. Gene fusion analysis is performed using Star-fusion and Arriba.

b) LOH: The heterozygosity of 45 SNVs are plotted and assessed across the 1p and 19q chromosomal regions as per Dubbink *et al* 2016[1]. Only 1p/19q LOH, LOH of chr 1, 7, 10, 11, 17, 19 and large-scale amplifications/deletions are assessed.

c) Variants are called against genome-build hg19 for the DNA and genome-build hg38 for the RNA. All nomenclature described using the Human Genome Variation Society (HGVS) guidelines (www.hgvs.org). Numbering starts with c.1 at the A of the ATG start codon. Variants are classified using the ACMG/AMP guidelines (Richards et al. 2015[2]) and ACGS best practice guidelines for variant classification in rare disease (www.acgs.uk.com/guidelines-for-variant-classification-v4-01-2020). Classification of variants identified during the course of genomic testing may change over time; where this change affects the clinical impact of a variant an amended report will be issued.

References:

1. Dubbink, Hendrikus J., Peggy N. Atmodimedjo, Johan M. Kros, Pim J. French, Marc Sanson, Ahmed Idbaih, Pieter Wesseling et al. "Molecular classification of anaplastic oligodendroglioma using next-generation sequencing: a report of the prospective randomized EORTC Brain Tumor Group 26951 phase III trial." *Neuro-oncology* 18, no. 3 (2015): 388-400.

2. Richards, Sue, Nazneen Aziz, Sherri Bale, David Bick, Soma Das, Julie Gastier-Foster, Wayne W. Grody et al. "Standards and guidelines for the interpretation of sequence variants: a joint consensus recommendation of the American College of Medical Genetics and Genomics and the Association for Molecular Pathology." *Genetics in medicine* 17, no. 5 (2015): 405-423.
